# Development and Population Growth Rates of *Sitophilus zeamais* (Coleoptera: Curculionidae) Exposed to a Sublethal Concentration of Essential Oil of *Piper hispidinervum*

**DOI:** 10.3390/insects16070697

**Published:** 2025-07-06

**Authors:** Lucas Martins Lopes, Lêda Rita D’Antonino Faroni, Gutierres Nelson Silva, Douglas Rafael e Silva Barbosa, Marcela Silva Carvalho, Herus Pablo Firmino Martins, Thaís Rodrigues dos Santos, Igor da Silva Dias, Adalberto Hipólito de Sousa

**Affiliations:** 1 Center of Biological and Natural Sciences, Federal University of Acre, Rio Branco 69920-900, AC, Brazil; lucas.lopes@ifam.edu.br (L.M.L.); igor.dias@sou.ufac.br (I.d.S.D.); 2 Federal Institute of Amazonas, Eirunepé 69880-000, AM, Brazil; 3 Department of Agricultural Engineering, Federal University of Viçosa, Viçosa 36570-900, MG, Brazil; lfaroni@ufv.br; 4 Federal Institute of Mato Grosso do Sul, Nova Andradina 79750-000, MS, Brazil; gutierres.silva@ifms.edu.br; 5 Federal Institute of Maranhão, Codo 65400-000, MA, Brazil; douglas.barbosa@ifma.edu.br (D.R.e.S.B.); herus.pablo@acad.ifma.edu.br (H.P.F.M.); 6 Federal Institute of Mato Grosso do Sul, Naviraí 79950-000, MS, Brazil; marcela.carvalho@ifms.edu.br; 7 Faculty of Entomology and Biodiversity Conservation, Federal University of Grande Dourados, Dourados 79804-970, MS, Brazil; thais_rodrigues260@hotmail.com

**Keywords:** corn weevil, safrole, reproduction, alternative control

## Abstract

Essential oils have emerged as promising alternatives for pest insect control. However, sublethal effects on insect reproduction and development are rarely explored, despite their relevance to integrated pest management (IPM). This study evaluated the sublethal effects of *Piper hispidivervum* C. DC. essential oil (EOPH) on the development and population growth of four populations of *Sitophilus zeamais* Motschulsky (Coleoptera: Curculionidae), as well as the persistence of safrole residue in treated corn grains. Population development rates were determined using emergence curves and total emerged adults, while population growth was assessed by counting live insects in the feeding substrate at different storage intervals. Safrole residue persistence was analyzed using solid-phase microextraction in headspace mode (SPME-HS). Sublethal exposure to EOPH significantly reduced the development rate, total emergence, and growth in three of the four populations. The population from Crixás, GO, showed no significant reduction, with a population curve overlapping the control. The lethal dose was reduced by 98.20%, indicating low persistence and potential food safety. The EOPH exhibited sublethal effects on *S. zeamais* populations, reducing both development rates and population growth. This reduction varied among the populations studied. Further research is encouraged to explore its effects on different insect populations and under broader environmental conditions.

## Simple Summary

Insects that damage stored grains, such as corn, cause major losses for farmers and food producers around the world. Finding safer and more sustainable ways to control these pests is a growing concern. In this study, we tested the effect of a sublethal concentration of essential oil extracted from a plant called *Piper hispidinervum* on a common grain pest known as the maize weevil. We observed whether this plant oil, even at doses that do not kill the insects immediately, could affect their ability to grow and reproduce over time. We tested four different insect populations and also checked how long a natural compound from the oil remained in the corn after treatment. The results showed that the essential oil reduced the number of insects that developed in most populations. One population, however, was not strongly affected. We also found that the only safrole that was studied in the oil broke down quickly, leaving only a very small amount after some time, which is good news for food safety. The results highlight the potential of plant-based essential oils to complement the existing pest control methods in stored grains, promoting safer and more sustainable management practices.

## 1. Introduction

The occurrence of pest insects during storage is one of the main causes of qualitative and quantitative loss of grains. The maize weevil, *Sitophilus zeamais* (Motschulsky, 1885) (Coleoptera: Curculionidae), is the main pest of stored corn in the world [1]. The control of this insect is carried out through the application of pyrethroid insecticides, organophosphates, and the fumigant phosphine (PH_3_) [2,3]. However, due to the high levels of resistance to these insecticides [4,5,6], studies on alternative methods for the control of stored grain pests have been widely disseminated, such as the use of botanical insecticides. The use of essential oils represents an environmentally friendly approach and a potentially cost-effective alternative for pest management [7].

Botanical insecticides are used for the alternative control of various pests in many countries [8]. They are normally used in the form of powders, extracts, or oils, are easy to obtain, and are generally harmless to the applicator and consumers [9]. Essential oils are derived from the secondary metabolism of aromatic plants, characterized by a complex of compounds, and their constituents are a promising alternative for the protection of stored products [10]. Essential oils and certain compounds have been shown to cause insect mortality through both contact and fumigation mechanisms [11].

*Piper hispidinervum* C. DC., 1917, also known as long pepper, is an aromatic plant endemic to the Amazon that has great potential in essential oil production [12,13]. The essential oil of *P. hispidinervum* is rich in safrole, a phenylpropanoid used in the chemical industry for the synthesis of heliotropin (fragrance fixative) [14] and piperonyl butoxide—PBO (insecticide synergist) [15]. In addition, the insecticidal effects of the essential oil of *P. hispidinervum* (EOPH) on stored grain pest insects are reported by several authors, such as toxicity (fumigant and contact) [16,17], repellency [18], reduction in development rates [19], and residual effect [20,21].

Most investigations into the insecticidal effect of essential oils on grain pests focus on toxicity and repellency [22]. However, the sublethal effects on reproductive patterns, such as development rates and population growth, are often neglected. Stored product pests, when sublethally exposed to synthetic or botanical insecticides, may exhibit not only harmful effects but, under certain circumstances, also show positive responses in their physiology and behavior [23,24]. These responses are called hormesis, and although the mechanisms that explain these responses (beneficial or harmful) are not yet well-understood, it has been described that individuals sublethally exposed to insecticides show changes in relevant characteristics such as development time, longevity, fertility, fecundity, locomotion, sexual communication, oviposition, and feeding [25,26,27,28].

Investigating the sublethal effects of botanical insecticides on reproductive patterns is essential to avoid failures in the control of stored grain pests, which result in the emergence of resistant populations or outbreaks of secondary pests [28]. Given the above, the objective of this study was to evaluate the sublethal effect of the essential oil of *P. hispidivervum* on the development and population growth rates of four Brazilian populations of *S. zeamais*. Additionally, the persistence of safrole residue in corn grains treated with *P. hispidivervum* oil was investigated.

## 2. Materials and Methods

The bioassays were conducted at the Integrated Pest Management Laboratory and at the Natural Products, Microbiology, and Biotechnology Laboratory at the Universidade Federal do Acre—Rio Branco campus and at the Ecotoxicology and Stored Grain Integrated Pest Management Laboratories at the Universidade Federal de Viçosa—Viçosa campus.

### 2.1. Populations of Sitophilus zeamais

Four Brazilian populations of *S. zeamais* collected in the municipalities of Cristalina (GO), Crixás (GO), Jacarezinho (PR), and Juiz de Fora (MG) were used in this study. The population of Cristalina was considered the standard of susceptibility based on the toxicological tests conducted by Lopes et al. [17].

The insects were raised in 1.5 L glass jars under constant temperature (27 ± 2 °C), relative humidity (70 ± 5%), and scotophase (24 h). Corn grains were used as a food substrate, with a moisture content of 13% on a wet basis (w.b.), previously fumigated with phosphine (PH_3_) (Gastoxin, Bernardo Química, São Vicente, SP, Brazil) and kept at −18 °C to prevent reinfestation.

### 2.2. Obtaining and Extracting the Essential Oil

*Piper hispidinervum* samples (leaves) were collected in the rural area of the municipality of Bujari, Acre (AC), Brazil (9°58′29″ S, 67°48′36″ W). Bujari is located 196 m above sea level and 23 km northwest of Rio Branco, the capital and largest city of the state. The plant material was harvested in the morning, in June 2017, and the essential oil was extracted from the collected leaves. A specimen was deposited at the UFACPZ Herbarium of the Federal University of Acre in Rio Branco under registration number UFACPZ 20.647. The material was identified by Dr. Elsie Franklin Guimarães, who works at the Herbarium of the Botanical Garden of Rio de Janeiro (Herbário RB, Rio de Janeiro, Brazil).

All botanical material was collected with the consent of the Brazilian Ministry of Environment and Climate Change (MMA), abiding by Biodiversity Law No. 13.123/2015 and Decree No. 8.772/2016. The former addresses genetic heritage, protection, and diligent access to local traditional knowledge and conservation and sustainable use of biodiversity. Decree No. 8.772/2016 regulates the Biodiversity Law and created the National System for the Management of Genetic Heritage and Associated Traditional Knowledge (SisGen electronic system). It is worth noting that Dr. Adalberto Hipólito de Sousa obtained permission from SisGen (No. ADEFA9D) to collect botanical material from *P. hispidinervum*. The experiment with wild *P. hispidinervum*, including the collection of plant material, complied with the relevant institutional, national, and international guidelines and legislation.

The *Piper hispidinervum* essential oil (EOPH) was extracted by hydrodistillation using a Clevenger-type apparatus coupled to a 5 L volumetric flask and a heating mantle (0321a28, Quimis, Diadema, SP, Brazil). The EOPH was separated from the emulsion via decantation in a separation funnel, and anhydrous sodium sulfate was used for the analysis (Synth, Diadema, SP, Brazil). The essential oil obtained was stored inside an amber flask at 4 ± 1 °C.

### 2.3. Essential Oil Composition

The EOPH was analyzed using gas chromatography combined with mass spectrometry (GC–MS) using a QP2010 system (Shimadzu, Kyoto, Japan). The chromatographic conditions included using a fused-silica capillary column (30 m length, 0.25 mm internal diameter) with an RTX^®^-5MS stationary phase (0.25 µm film thickness) and helium as the carrier gas applied at a flow rate of 1.2 mL min^−1^. The temperature at the injector was 220 °C, and the column was initially at 60 °C. The heating rate was set to increase by 2 °C min^−1^ up to 200 °C, and then by 5 °C min^−1^ up to 250 °C, remaining in this condition for 1 min. The mass spectra were obtained via electron impact at 70 eV, with range coverage from 29 to 400 (m/z). The chromatograph operated in the full-scan mode, with a split ratio of 1:20. The total analysis time was 81 min.

The compounds were identified by comparing the resulting mass spectra with those listed in the NIST library and by visual interpretation [29]. They were confirmed using the Kovats index (KI) and by comparison with the literature data (49451-U, Supelco, Bellefonte, PA, USA). The KI value of each compound was calculated based on the retention time of the compound and on the alkanes of the standard alkane solution. The relative percentage of each compound corresponded to the ratio between the area of each peak and the total area of all sample components.

### 2.4. Absolute Quantification of Safrole

The absolute quantification of safrole present in the EOPH was determined using a gas chromatograph with a flame ionization detector (GC/FID) (GC2014, Shimadzu, Japan). Five concentrations of safrole (Safrole solution, Supelco, P.A., Darmstadt, Germany), 0.25, 0.50, 1.00, 1.50, and 2.00 mg mL^−1^, diluted in methanol, in three replicates, were injected. The EOPH was also injected at a concentration of 1 mg mL^−1^ diluted in methanol (Vetec, UV/HPLC 99.9%, Verden, Germany) in triplicates.

The chromatographic conditions for safrole quantification were as follows: 30 cm long capillary column (DB-5, Shimadzu, Japan) with 0.25 mm internal diameter and 0.10 µm film thickness, nitrogen (Air Products, U.P. 99.999%, São Paulo, Brazil) as the carrier gas with a flow of 1.82 mL min^−1^, injector temperature of 220 °C, flame ionization detector temperature of 300 °C, and a 1:5 split ratio. The initial column temperature was 60 °C, with a ramp rate of 5 °C min^−1^ up to 120 °C; this temperature was held for 1 min. The total analysis time was 12 min.

### 2.5. Population Development Rates

The bioassays were conducted in glass flasks with a capacity of 0.8 L and breathable lids, containing 200 g of corn, with a moisture content of 13% (w.b.), exposed to a sublethal concentration of the EOPH, 85.42 µL kg^−1^ (CL_30_ of the Cristalina population, GO). The LC_30_ value was estimated based on the lethal concentration data. Concentration–response curves were obtained through bioassays using increasing concentrations of the essential oil. Mortality data were subjected to probit analysis using the PROBIT procedure in SAS software (SAS Institute, 9.0, Minato, Japan) [30], thereby generating concentration–mortality curves.

The essential oil was previously diluted in acetone (analytical grade, ≥99%) to ensure proper homogenization and facilitate its application in the bioassays. The control was constituted by acetone. The EOPH and acetone were sprayed onto the grain mass using a dual-action airbrush with an internal mixing system and gravity feed (model BC 60, Steula, São Paulo, Brazil). The working pressure used in the spraying was 15 psi, and the volume of spray applied was 400 µL for every 200 g of corn. The essential oil was dispensed onto the grains using an automatic pipette, followed by manual shaking for two minutes to ensure homogeneous distribution.

The grains were infested with 50 unsexed adult insects from the four populations, aged one to three weeks. Subsequently, the vials were stored in BOD-type climate chambers under constant conditions of temperature (27 ± 2 °C), relative humidity (70 ± 5%), and scotophase (24 h). After 13 days, the insects were removed from the vials according to the method described by Trematerra et al. [31]. Four replicates were used for each population. The adult progeny obtained in the feeding substrate was counted and removed on alternate days from the first emergence.

The normalized accumulated emergence was initially analyzed in the populations. The sum of insect emergence was accumulated from the initial emergence and resulted in the sum of accumulated emergence (SEa, % day), calculated as follows: SEa = ∑SEd. The accumulated emergence data were analyzed because experimental errors are more likely when only the data from insect emergence evaluation on alternate days are considered, due to the influence of sampling times [31,32,33]. Additionally, the total number of emerged insects for the four populations exposed or not exposed to the EOPH was counted.

### 2.6. Population Growth and Grain Mass Loss

Population growth bioassays were performed using 0.8 L glass containers, each filled with 200 g of corn at 13% moisture content (w.b.), and treated with a sublethal dose of the EOPH (85.42 µL kg^−1^), corresponding to the CL_30_ determined for the Cristalina (GO) population [17]. The control was constituted by acetone. The EOPH and acetone were sprayed onto the grain mass using a dual-action airbrush with an internal mixing system and gravity feed (model BC 60, Steula, São Paulo, Brazil). The working pressure used in the spraying was 15 psi, and the volume of spray applied was 400 µL for every 200 g of corn.

The grains were infested with 50 unsexed adult insects from the four populations, aged one to three weeks. Subsequently, the vials were stored in BOD-type climate chambers under constant conditions of temperature (27 ± 2 °C), relative humidity (70 ± 5%), and scotophase (24 h). In this bioassay, the insects were not removed. Four replicates were used for each population. The number of live insects obtained in the feeding substrate was counted at 0, 30, 60, and 70 days from the start of the bioassays. Additionally, the mass loss of the grains was measured 70 days after the start of the tests. The percentage of grain mass loss (%) was calculated as the difference between the initial and final weights according to the following equation: ML = (Mi − Mf)/Mi × 100, where ML = mass loss (%), Mi = initial weight (g), and Mf = final weight (g).

### 2.7. Persistence of the Safrole Residue

The corn kernels were treated by contact with the EOPH at a concentration of 667.49 µL kg^−1^, which corresponds to a safrole concentration of 623.48 µg kg^−1^. The concentrations used corresponded to the lethal concentrations of the EOPH to kill 95% of the Cristalina population (LC_95_) [17]. The EOPH was sprayed with a dual-action airbrush, with an internal mixing system and gravity feed (model BC 60, Steula, São Paulo, Brazil). The working pressure used in the spraying was 15 psi, and the volume of spray applied was 400 µL for every 200 g of corn. The experimental units were composed of glass jars with a capacity of 0.8 L (8 cm in diameter × 15 cm in height) containing 200 g of corn.

After the corn treatment, the grains were kept for the 24 h exposure period. During this period, the jars were covered with organza fabric, which prevented insect escape while allowing gas exchange. After the exposure period, the grains were transferred to nylon bags (12 cm × 16 cm) and stored under constant temperature conditions (25 ± 2 °C) and relative humidity (70 ± 5%). The analysis of the safrole residue was performed using the optimized solid-phase microextraction method in headspace mode (SPME-HS/GC–FID), according to [34] during storage periods of 5, 10, 26, 60, and 90 days. The persistence of safrole was calculated using the calibration curve of the SPME-HS method. Five mathematical models (linear, quadratic, exponential, potential, and logarithmic) were tested to explain the degradation of the safrole residue. The choice of the model was made based on the coefficient of determination (R^2^), the estimate of the root mean square error (RMSE) (closer to zero), and the significance (*p* < 0.05).

### 2.8. Statistical Analyses

The normalized accumulated emergence data, daily emergence, and number of live insects were subjected to non-linear regression analyses using the curve-fitting procedure of the SigmaPlot software, version 13.1 (Systat Software, Inc., San Jose, CA, USA). The total number of emerged insects and grain mass loss were subjected to analysis of variance [30].

Subsequently, tests were conducted to compare means between the populations (*p* < 0.05) and *F*-tests between treatments with and without the essential oil (*p* < 0.05) [30]. The persistence curves of the safrole residue in corn were subjected to regression analysis using the curve-fitting procedure in SigmaPlot, version 13.1 (Systat Software, Inc., San Jose, CA, USA).

## 3. Results

### 3.1. Composition of the EOPH

Six constituents were identified in the EOPH through chromatographic analysis (Table 1). Safrole is the major compound, accounting for 93.0% of the identified compounds, followed by bicyclogermacrene, pentadecane, spathulenol, p-cymen-8-ol, and (E)-caryophyllene, with 2.05%, 1.60%, 1.46%, 1.20%, and 0.69%, respectively. The absolute concentration of safrole in the EOPH was determined through GC–FID according to the retention time of safrole P.A., where it was found that safrole represents 85% of the essential oil.

### 3.2. Population Development Rates

**Figure 1:**
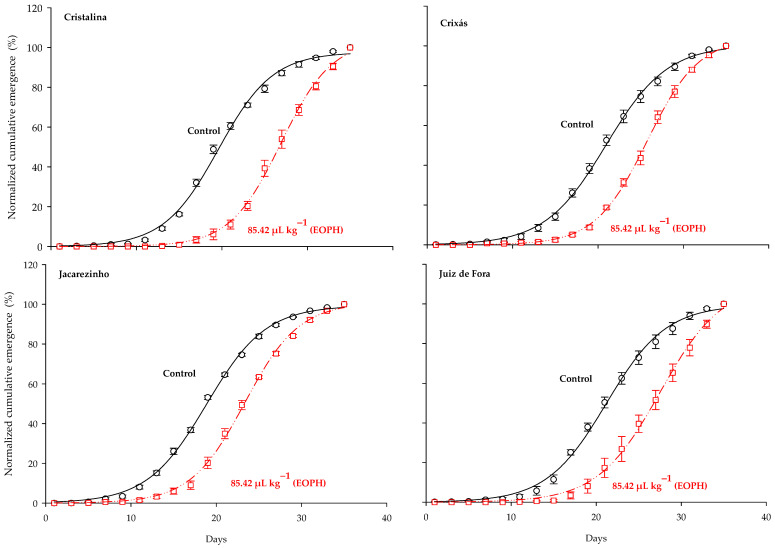
Normalized accumulated emergence (%) of the four populations of *S. zeamais* not exposed (control) and exposed to a sublethal concentration of the EOPH (CL_30_ = 85.42 µL kg^−1^).

The three-parameter sigmoid model y = *a*/(1 + exp(−(x − *b*)/*c*)) was the one that fitted the normalized cumulative emergence of adults from the populations of *S. zeamais* (*p* < 0.0001; R^2^ = 0.99; Table 2 and Figure 1). Significant differences were observed between the cumulative emergence curves of populations exposed to a sublethal concentration of the EOPH, with no overlap occurring in relation to the control. In general, all estimates of the inflection points (parameter b) were higher in the populations exposed to the EOPH, suggesting a later emergence of insects (Table 2 and Figure 1). The variations of parameter b in the populations of Cristalina (GO), Juiz de Fora (MG), Jacarezinho (PR), and Crixás (GO) in relation to the control were 33%, 30%, 24%, and 23%, respectively.

**Figure 2:**
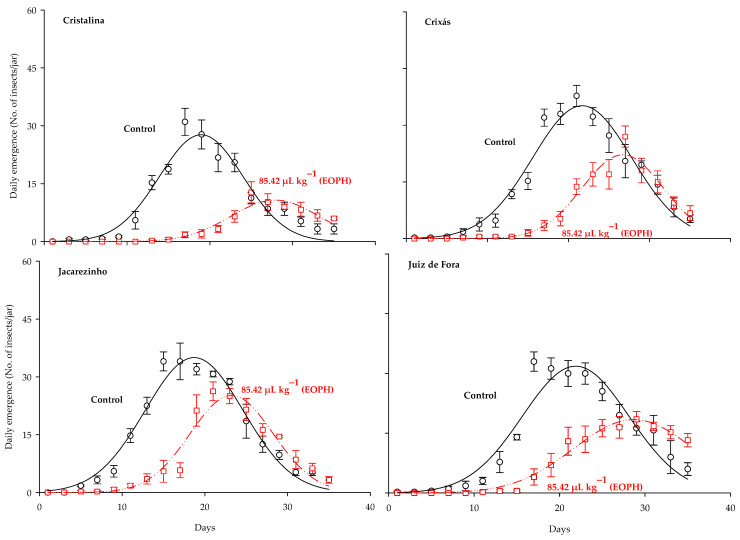
Daily emergence of the four populations of *S. zeamais* not exposed (control) and exposed to a sublethal concentration of the EOPH (CL_30_ = 85.42 µL kg^−1^).

The three-parameter Gaussian model *y* = *a*exp(−0.5((x−*b*)/*c*)^2^) was the best fit for the daily emergence of *S. zeamais* populations (*p* < 0.0001; R^2^ > 0.91; Table 2 and Figure 2). Significant differences were observed between the daily emergence curves of the populations exposed to a sublethal concentration of the EOPH, with no overlap occurring in relation to the control. Estimates for the maximum peak of daily emergence (parameter a) were lower in all populations exposed to the EOPH, showing a lower emergence of adult insects (Table 2 and Figure 2). The variations of parameter a in the populations of Cristalina (GO), Juiz de Fora (MG), Crixás (GO), and Jacarezinho (PR) in relation to the control were 61%, 42%, 37%, and 28%, respectively.

Estimates on the days needed to reach the maximum emergence peak (parameter b) were higher in the populations exposed to the EOPH, showing that the occurrences of population peaks were later compared to the control (Table 2 and Figure 2). The variations of parameter b in the populations of Cristalina (GO), Juiz de Fora (MG), Jacarezinho (PR), and Crixás (GO) in relation to the control were 47%, 31%, 24%, and 23%, respectively.

**Figure 3:**
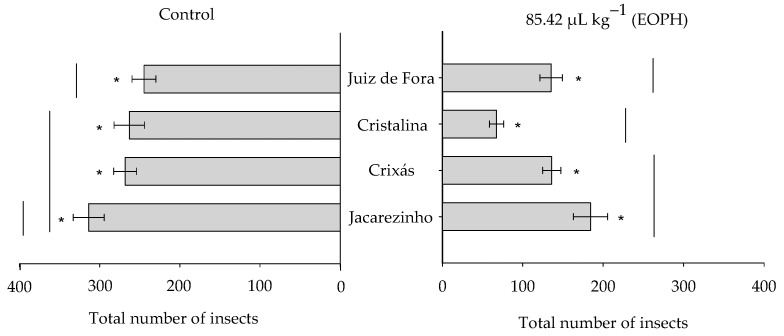
Total number of emerged insects from the four populations of *S. zeamais* not exposed (control) and exposed to a sublethal concentration of the EOPH (CL_30_ = 85.42 µL kg^−1^). The “*” indicate a significant difference between the population exposed and not exposed to OEPH according to the F-test (*p* < 0.05).

The total number of emerged insects varied significantly among the populations of *S. zeamais* (F_3;24_ = 9.81; *p* < 0.01) between the EOPH treatment and control (F_1;24_ = 159.31; *p* < 0.01), but there was no significant interaction between these two factors (F_3;24_ = 2.77; *p* = 0.06) (Figure 3). Overall, the total number of emerged insects was lower in the populations exposed to the EOPH compared to the control. The population of Cristalina, GO, showed the lowest number of emerged insects (67.25 ± 8.93) when compared to the other populations exposed to the EOPH (Figure 3).

### 3.3. Population Growth and Grain Mass Loss

**Figure 4:**
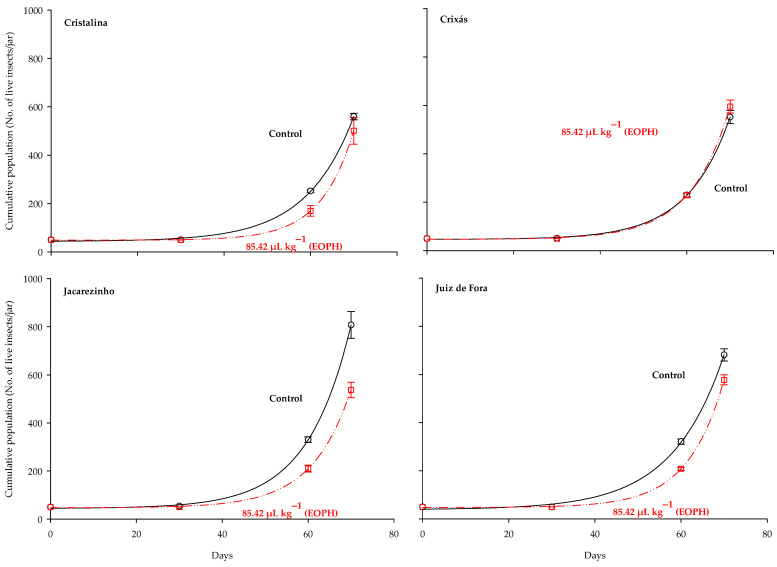
Population growth of the four populations of *S. zeamais* not exposed (control) and exposed to a sublethal concentration of the EOPH (CL_30_ = 85.42 µL kg^−1^).

The three-parameter exponential growth model *y = a + bexp^(cx)^* was the best fit for the population growth (live insects) of the *S. zeamais* populations (*p* < 0.05; R^2^ = 0.99; Table 3 and Figure 4). Substantial variations were observed in the population growth curves of the populations exposed to a sublethal concentration of the EOPH, with no overlap with the control, except for the population of Crixás, GO (Figure 4). The populations of Cristalina (GO), Jacarezinho (PR), and Juiz de Fora (MG) experienced a reduction in population growth similar to the rates of population development.

**Figure 5:**
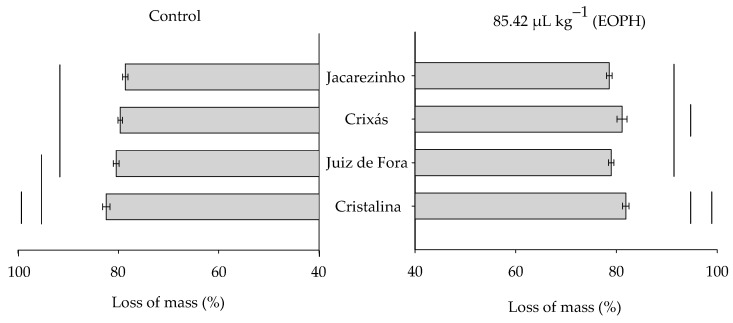
Grain mass loss (%) at 70 days of the four populations of *S. zeamais* not exposed (control) and exposed to a sublethal concentration of the EOPH (CL_30_ = 85.42 µL kg^−1^).

The grain mass loss at 70 days varied significantly among the populations of *S. zeamais* (F_3;24_ = 10.37; *p* < 0.01), but there was no variation between the treatment with the EOPH and the control (F_1;24_ = 0.08; *p* = 0.79) or in the significant interaction between these factors (F_3;24_ = 1.80; *p* = 0.17) (Figure 5).

### 3.4. Safrole Residue

**Figure 6:**
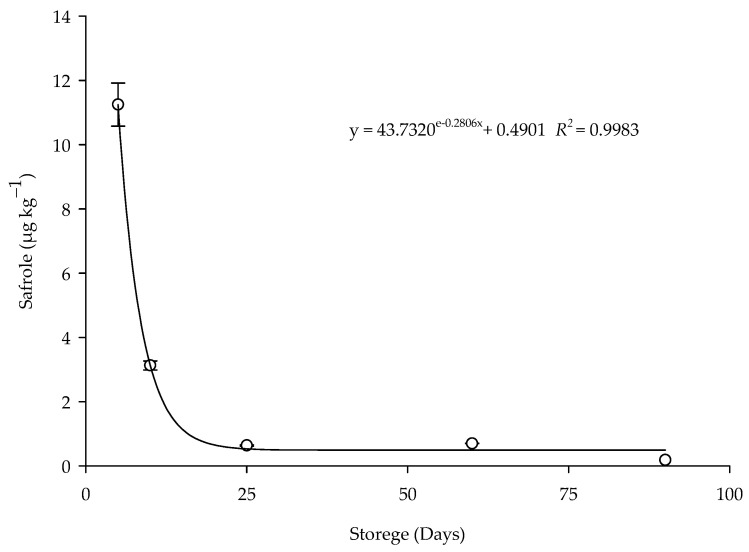
Persistence of safrole residue in the corn treated with the EOPH (LC_95_) by contact during storage.

The degradation of the safrole residue in the corn grains treated with the EOPH (LC_95_) by contact during the storage period was explained by different adjusted regression mathematical models (Table 4). In the treated grains, the adjusted model was exponential (y = ae − bx + c) (Figure 6), with the R^2^ value of 0.9983, RMSE of 0.1723, and *p* of 0.0017. The concentrations detected for the grains treated by contact were 11.2441 µg kg^−1^ on the fifth day and 0.1833 µg kg^−1^ at 90 days. A reduction of 98.20% in the concentration of safrole present in the grains was observed after five days of storage.

## 4. Discussion

This pioneering study is the first to compile data on the sublethal effects of the *P. hispidivervum* essential oil on the development and population growth of four populations of *S. zeamais*. Moreover, it stands out for investigating the persistence of safrole residues in treated maize grains, providing valuable insights into the safety and effectiveness of this product for pest management in stored grains.

The EOPH is rich in safrole, which represents 93.0% of the compounds identified, as revealed by chromatographic analysis (GC–MS). Safrole has already been reported as the major compound in the essential oil of *P. hispidinervum*, with concentrations greater than 90% in the Amazon region [35] or between 77% to 90% in plants cultivated in southern Brazil [36,37,38]. The other minor compounds identified in the EOPH were also reported by other authors, such as pentadecane [38], bicyclogermacrene and (E)-caryophyllene [13,16,38], spathulenol [18,39], and p-cymen-8-ol [13,18]. The detection of only six compounds in the EOPH can be explained by the high predominance of safrole, which hampers the identification of minor compounds; by the influence of the extraction and processing methods [40]; and by the genetic and environmental variability among plant populations [41].

The bioinsecticidal activity of the EOPH on stored grain pests has already been described in the literature [16,17,19,21], being mainly attributed to the action of its compounds. Safrole primarily acts as a neurotoxin, disrupting the insect nervous system. This interference can impair essential neurophysiological processes, ultimately leading to insect mortality [42]. In laboratory assays, safrole exhibits significant neurotoxic effects on insects, such as *S. zeamais*, leading to disorientation, paralysis, and ultimately death [34].

Beyond assessing toxicity, it is crucial to understand the sublethal effects of agents used for pest insect control. Such in-depth knowledge supports the development of more effective and sustainable management strategies in stored grains. Sublethal exposure to the EOPH (CL_30_ = 85.42 µL kg^−1^) negatively influenced the population development rates and the total number of insects that emerged from the four Brazilian populations of *S. zeamais*.

The lowest daily emergence values, the total emerged insects, and the longest time required for the occurrence of the population peak among the four populations led to a lower normalized cumulative emergence and lower population growth of these populations, except for the population of Crixás, GO, which showed an overlap of the population growth curve with the control. One explanation for the overlapping population growth curves observed in the Crixás population is the phenomenon of hormesis, in which exposure to a sublethal dose of an insecticide stimulates physiological mechanisms that enhance insect survival, reproduction, or overall performance [25]. Moreover, this overlap may be associated with the genetic variability that exists among insect populations from different geographic regions. It is possible that the Crixás population harbors alleles that confer greater metabolic tolerance to safrole (the main compound in the *P. hispidivervum* essential oil) compared to the other populations evaluated [43]. Negative effects of sublethal exposure of insect populations to essential oils on biological characteristics, such as development rates, population growth, and total emergence of stored grain pest insects, have already been reported by several authors [5,8,22,24].

Although sublethal exposure to the EOPH significantly reduces the development of insect populations in the grain storage industry, it cannot completely eradicate them. Sublethal exposure to synthetic or botanical insecticides can cause changes in insect homeostasis, thus allowing their survival [39]. Although the damage caused to pests is maintained for several generations, the intensity of selection pressure could contribute to the emergence of resistant insect populations [8,33,44].

Some insecticides can modulate the activity not only of detoxification enzymes in living insects, but also of enzymes responsible for digestive and energy metabolism. Enhanced detoxification activity is often associated with insecticide resistance, but the activity of digestive and energy metabolism enzymes may also be important in mitigating the adaptive costs generally associated with insecticide resistance [39,45]. A strategy to prevent the emergence of resistant biotypes is the combination of various control methods [46]. Safrole acts not only as a bioactive compound but also as a precursor of piperonyl butoxide, a well-known synergist that enhances the effectiveness of insecticides by inhibiting detoxification enzymes in insects, thereby increasing their control efficacy.

In this way, the results of this study indicate that the EOPH can be considered a potential bioinsecticide for the control of *S. zeamais* and can be included in strategies to minimize the probability of developing resistance to synthetic insecticides. It is worth noting that the use of different insecticides for which insects do not show cross-resistance can substantially contribute to reducing the speed of resistance development [32,47]. Thus, the rotational use of insecticides can minimize the emergence of resistant genotypes and place individuals at an adaptive disadvantage [8]. In this strategy, sublethal concentrations of the *Piper* oil could be used in rotation to diversify selection pressures, as plant-derived compounds often act on multiple molecular targets or disrupt insect behavior. This contrasts with the single-site action of many synthetic insecticides, reducing the likelihood of cross-resistance and helping to delay resistance evolution in pest populations.

The decrease in the persistence of the safrole residue with the extension of the corn storage period was evident, with a reduction of 98.20% after five days of storage. The concentrations of safrole used in the treatment of corn were lower than the concentration limits allowed by ANVISA (1.00 mg kg^−1^; Resolution (RDC) No. 2 of 15 January 2007) and the European Union (1.00 mg kg^−1^; Resolution (EC) No. 1334 of 16 December 2008) for beverages and food. The residual effect of the EOPH has already been analyzed on *S. zeamais* in corn grains during the 120-day storage period, and a low residual effect was found, with mortality below 17.5% after 30 days of storage [48]. The low persistence of botanical insecticides in stored products has already been reported in the literature [20,49]. The rapid degradation of essential oils is explained by the chemical instability of their constituents due to oxidation, isomerization, cyclization, or dehydrogenation reactions that are triggered by enzymes or other molecules, such as oxygen [50,51]. Temperature, light, and the availability of atmospheric oxygen also influence the stability of essential oils [52].

The reduction in development rates and population growth of populations exposed to a sublethal concentration of the EOPH demonstrates the bioinsecticidal activity of this oil. Application of oils on already infested grains, including grains harvested with hidden infestations from the field, could help limit the emergence and development of new pest generations. By affecting insect behavior and survival, even at sublethal levels, these oils may reduce postharvest damage and slow population growth during storage. The insecticidal effect of the *Piper* oil is likely due to a combination of direct adult toxicity, oviposition deterrence, and inhibition of offspring emergence. These effects are probably associated with bioactive compounds that may interfere with the insect nervous and/or reproductive systems. Although these mechanisms were not directly tested in this study, their multifaceted action could reduce pest population growth and enhance control efficacy.

Understanding the sublethal effects of synthetic or natural insecticides should always be a focus in toxicological studies, as sublethal exposures of insects are likely more frequent than lethal ones in storage environments [39]. Regardless of the type of insecticide used, lethal and sublethal effects must be fully explored, and this information should be used in integrated pest management decision-making.

The application of sublethal doses of the EOPH can be integrated into more sustainable management strategies against *S. zeamais*, reducing reliance on synthetic insecticides. The rapid degradation of the oil and its major component, safrole, suggests a lower risk of persistent residues, making it safer for consumption. To enhance its effectiveness, future studies should evaluate possible organoleptic changes in the treated grains and investigate the effects on already infested grains, including under field conditions. These aspects are crucial to validate the practical application of this essential oil in storage and agricultural production systems.

The EOPH exhibited sublethal effects on the *S. zeamais* populations, reducing both development rates and population growth. This reduction varied among the populations studied. Further research is encouraged to explore its effects on different insect populations and under broader environmental conditions.

## Figures and Tables

**Table 1:** Chemical composition and relative concentrations of the compounds identified in the EOPH using gas chromatography–mass spectrometry (GC–MS) analysis.

Constituent	RI^1^ Literature	RI^1^ Calculated	%Relative
Safrole	1285	1292	93.00
Bicyclogermacrene	1500	1493	2.05
Pentadecane	1500	1498	1.60
Espathulenol	1577	1573	1.46
p-Cimen-8-ol	1179	1184	1.20
(E)-caryophyllene	1417	1415	0.69

RI^1^ = retention index.

**Table 2:** Summary of non-linear regression analyses of the population development curves of *Sitophilus zeamais* under sublethal exposure to the EOPH.

Variable (Figure)	Model	Pop *	Treatment	Parameter Estimates (±SEM) **			df_error_	*F*	*R^2^*
				*a*	*b*	*c*			
NCE (%) (Figure 1)	y = *a*/(1 + exp(−(x−*b*)/*c*))	CA	Control	197.64 ± 1.43	19.59 ± 0.20	3.19 ± 0.17	15	2562.50	0.99
			85.42 µL kg^−1^	103.89 ± 2.16	26.01 ± 0.19	2.99 ± 0.13	15	4601.12	0.99
		CS	Control	100.37 ± 1.11	20.95 ± 0.15	3.58 ± 0.12	15	6310.82	0.99
			85.42 µL kg^−1^	104.28 ± 1.06	25.76 ± 0.10	3.04 ± 0.07	15	14,632.46	0.99
		JO	Control	99.34 ± 0.83	18.85 ± 0.12	3.48 ± 0.10	15	7682.27	0.99
			85.42 µL kg^−1^	100.86 ± 1.23	23.35 ± 0.14	3.19 ± 0.11	15	6658.73	0.99
		JF	Control	99.55 ± 1.64	21.19 ± 0.22	3.52 ± 0.17	15	2925.45	0.99
			85.42 µL kg^−1^	110.84 ± 3.40	27.51 ± 0.31	3.68 ± 0.17	15	3694.30	0.99
DE (Figure 2)	y = *a*exp(−0.5((x−*b*)/*c*)^2^)	CA	Control	27.67 ± 1.63	18.97 ± 0.34	5.07 ± 0.34	15	98.92	0.93
			85.42 µL kg^−1^	10.73 ± 0.68	27.87 ± 0.41	5.35 ± 0.45	15	104.42	0.93
		CS	Control	35.15 ± 1.49	21.69 ± 0.30	6.13 ± 0.30	15	170.60	0.96
			85.42 µL kg^−1^	22.17 ± 1.10	26.70 ± 0.29	5.00 ± 0.30	15	164.74	0.96
		JO	Control	35.01 ± 1.28	18.71 ± 0.25	5.94 ± 0.25	15	231.13	0.97
			85.42 µL kg^−1^	25.16 ± 1.39	23.25 ± 0.32	5.03 ± 0.32	15	122.07	0.94
		JF	Control	31.85 ± 1.92	21.89 ± 0.43	6.23 ± 0.44	15	84.29	0.92
			85.42 µL kg^−1^	18.42 ± 0.57	28.68 ± 0.33	7.02 ± 0.36	15	380.58	0.98

Note: * Pop, population; NCE, normalized cumulative emergence; DE, daily emergence; CA, Cristalina; CS, Crixás; JO, Jacarezinho; JF, Juiz de Fora. ** All parameter estimates were significant at *p* < 0.01 (Student’s *t*-test), and all the models were significant at *p* < 0.01 (Fisher’s *F*-test).

**Table 3:** Summary of non-linear regression analyses of the population growth curves of *Sitophilus zeamais* under sublethal exposure to the EOPH.

Variable (Figure)	Model	Pop	Treatment *	Parameter Estimates (±SEM) **			df_error_	*F*	*R^2^*
				*a*	*b*	*b*			
CP (Figure 4)	y = *a* + *b*exp^(*c*x)^	CA	Control	43.51 ± 7.11	10.85 ± 0.32	0.09 ± 0.01 *	1	1088.47	0.99
			85.42 µL kg^−1^	48.49 ± 1.56 *	0.04 ± 0.01	0.13 ± 0.00 *	1	1494.40	0.99
		CS	Control	46.25 ± 3.25	0.40 ± 0.10	0.10 ± 0.00 *	1	3182.00	0.99
			85.42 µL kg^−1^	46.50 ± 3.70	0.24 ± 0.06	0.11 ± 0.00 *	1	4115.95	0.99
		JO	Control	44.32 ± 5.90	0.78 ± 0.19	0.10 ± 0.00 *	1	3297.52	0.99
			85.42 µL kg^−1^	46.86 ± 3.32 *	0.21 ± 0.05	0.11 ± 0.00 *	1	4082.61	0.99
		JF	Control	38.82 ± 12.66	1.86 ± 0.91	0.08 ± 0.00 *	1	572.54	0.99
			85.42 µL kg^−1^	47.68 ± 2.43 *	0.12 ± 0.02	0.13 ± 0.00 *	1	8765.08	0.99

Note: * Pop, population; Tre, treatment; CP, cumulative population; CA, Cristalina; CS, Crixás; JO, Jacarezinho; JF, Juiz de Fora. ** All parameter estimates were significant at *p* < 0.01 (Student’s *t*-test), and all the models were significant at *p* < 0.01 (Fisher’s *F*-test).

**Table 4:** Adjusted mathematical models for safrole residue in the corn treated with the essential oil of *P. hispidinervum* by contact during storage.

Treatment	Model	Equation	*R^2^*	RQEM	*p*
Contact	Linear	y = −0.0845x + 6.3884	0.4309	3.1405	0.2288
	Quadratic	y = 0.0029x^2^ − 0.3559x + 9.4685	0.6563	2.4406	0.3437
	Exponential	y = 43.7320e^−0.2806x^ + 0.4901	0.9983	0.1723	0.0017
	Potential	y = 442.664 (1 + x)^−2.0510^	0.9954	0.2834	0.0001
	Logarithmic	y = −0.9699ln (x − 4.9992) + 4.3262	0.9902	5.0454	0.0098

## Data Availability

The raw data supporting the conclusions of this article will be made available by the authors upon request.
